# Bidirectional Interactions between Green Tea (GT) Polyphenols and Human Gut Bacteria

**DOI:** 10.4014/jmb.2306.06014

**Published:** 2023-07-12

**Authors:** Se Rin Choi, Hyunji Lee, Digar Singh, Donghyun Cho, Jin-Oh Chung, Jong-Hwa Roh, Wan-Gi Kim, Choong Hwan Lee

**Affiliations:** 1 Department of Bioscience and Biotechnology, Konkuk University, Seoul 05029, Republic of Korea; 2 Amorepacific R&I Center, Yonggu-daero, Yongin, Republic of Korea; 3 Research Institute for Bioactive-Metabolome Network, Konkuk University, Seoul 05029, Republic of Korea

**Keywords:** Gut microbiota, green tea, polyphenols, biotransformation, LC-MS/MS

## Abstract

Green tea (GT) polyphenols undergo extensive metabolism within gastrointestinal tract (GIT), where their derivatives compounds potentially modulate the gut microbiome. This biotransformation process involves a cascade of exclusive gut microbial enzymes which chemically modify the GT polyphenols influencing both their bioactivity and bioavailability in host. Herein, we examined the in vitro interactions between 37 different human gut microbiota and the GT polyphenols. UHPLC-LTQ-Orbitrap-MS/MS analysis of the culture broth extracts unravel that genera *Adlercreutzia*, *Eggerthella* and *Lactiplantibacillus plantarum* KACC11451 promoted C-ring opening reaction in GT catechins. In addition, *L. plantarum* also hydrolyzed catechin galloyl esters to produce gallic acid and pyrogallol, and also converted flavonoid glycosides to their aglycone derivatives. Biotransformation of GT polyphenols into derivative compounds enhanced their antioxidant bioactivities in culture broth extracts. Considering the effects of GT polyphenols on specific growth rates of gut bacteria, we noted that GT polyphenols and their derivate compounds inhibited most species in phylum Actinobacteria, Bacteroides, and Firmicutes except genus *Lactobacillus*. The present study delineates the likely mechanisms involved in the metabolism and bioavailability of GT polyphenols upon exposure to gut microbiota. Further, widening this workflow to understand the metabolism of various other dietary polyphenols can unravel their biotransformation mechanisms and associated functions in human GIT.

## Introduction

The human microbiome, consisting of a wide array of microorganisms, exerts both direct and indirect influences on numerous physiological functions. It is primarily characterized by the dominance of various bacterial phyla including Firmicutes, Bacteroidetes, Actinobacteria, Proteobacteria, and Verrucomicrobia developed over the long period of evolution [1, 2]. These gut microbiota phyla influence important host functions like defense against pathogens, training host immune system, drug metabolism, toxin elimination, and nutrient assimilation [3]. The gut microbiome encodes approximately 40 times more genes than the host, enabling the catabolism of complex dietary compounds [4]. Therefore, the microbiome is considered an important factor that contributes to the interpersonal variations in response to diet. Hence, any imbalance in gut microbiota, also known as dysbiosis, results in various gastrointestinal disorders including inflammatory bowel disease (IBD), irritable bowel syndrome (IBS), and obesity with associated metabolic disorders [4]. In recent years, there has been a growing emphasis on importance of maintaining a balanced gut microbiota. Consequently, numerous studies have been carried out to promote and restore healthy gut microbiota, with particular attention given to utilization of prebiotics. In addition to the well characterized dietary fibers, polyphenols are also considered to have prebiotic functions owing to their bidirectional interaction with the gut microbiota [5, 6]. Certain polyphenols, including those found in green tea (GT), have been shown to promote growth and bioactivities of beneficial gut bacteria including Bifidobacteria and Lactobacilli [7].

**Fig. 1:**
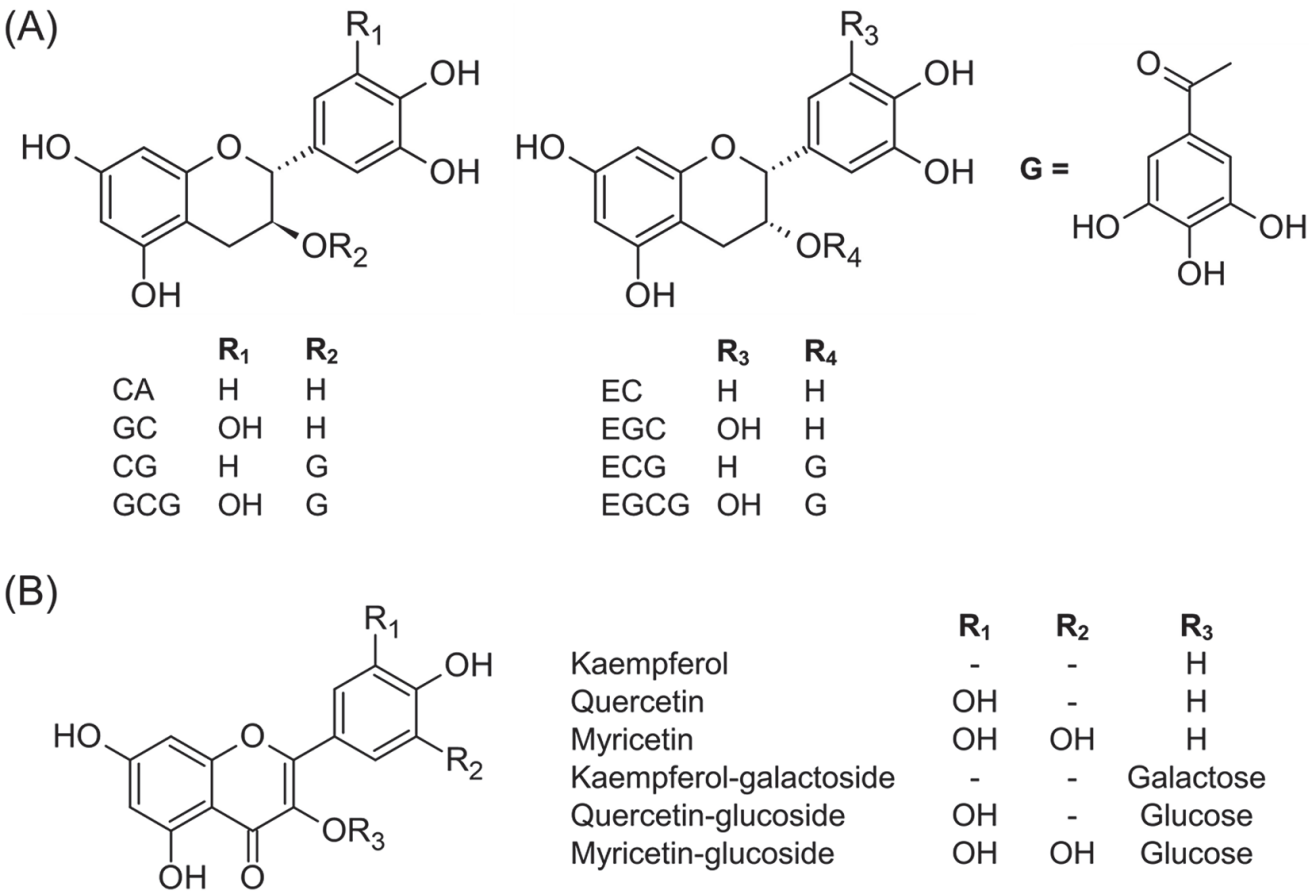
Chemical structures of major GT polyphenol classes used in this study. (**A**) GT catechins, and (**B**) flavonoids. Here, G represents gallate moiety.

Green tea (GT), a rich source of polyphenols, is among the most widely consumed beverage in the world and has recognized health benefits, in addition to its palatability. GT consumption has been reported to ameliorate certain metabolic disorders including cardiovascular diseases, obesity, and type 2 diabetes [8]. The health-promoting effects of major GT are generally attributed to the phenolic compounds (Fig. 1), such as catechins and flavonoids, with antioxidant, anti-aging, anti-tumor, and anti-microbial functions [8, 9]. Despite significant bioactivities of GT polyphenols, their low bioavailability in the small intestine poses challenge in understanding their clinical effects [10]. It has been suggested that GT metabolites, including polyphenols, are bio-transformed by the gut microbiota resulting in their increased bioavailability and in vivo functional effects [11, 12]. Recently, Chen *et al*.[11] and Rha *et al*. [13] showed that GT metabolites bio-transformed by intestinal microbes, such as C-ring cleaved derivative and flavonoid aglycone, have greater antioxidant activity compared to their precursor compounds. In addition, GT polyphenols and their bio-transformed derivatives are shown to modulate gut microbiota through promoting the growth of beneficial microbes [14-16]. An increasing number of studies are now exploring the effects of nutrient-derived and bio-transformed metabolites on the emergent community properties of gut microbiome.

The present study explores the bidirectional interactions between the GT polyphenols and gut microbiota to examine their bioavailability and prebiotic functions. However, the prebiotic effects of GT polyphenols are not conclusively proven as there are very limited studies which explains how the gut microbiota interact with GT compounds [17, 18]. Herein, we present an in vitro screening workflow to delineate the bidirectional interrelationship among the GT polyphenols, their biotransformation, and their growth modulatory effects on gut microbiota.

## Materials and Methods

### Chemicals and Reagents

High performance liquid chromatography (HPLC) grade ethanol, methanol, acetonitrile, and water were purchased from Fisher Scientific (USA). All analytical grade reagents used in this study were obtained from Sigma Chemical Co. (USA). Epigallocatechin gallate (EGCG), gallocatechin gallate (GCG), epicatechin gallate (ECG), catechin gallate (CG), epigallocatechin (EGC), gallocatechin (GC), epicatechin (EC), catechin (CA), gallic acid (G), pyrogallol, myricetin, quercetin, kaempferol, quercetin 3-glucoside, galactose, and glucose were purchased from Sigma (USA). Kaempferol-3-O-galactoside was purchased from ChemFaces Biochemical Co. Ltd. (China). The chemical structures of catechins and flavonoids used in this study are presented in Fig. 1.

### Preparation of Green Tea (GT) Polyphenol extracts

The dried green tea leaves (*Camelia sinensis* L. produced from Osulloc farm, Republic of Korea) were first extracted with 70% ethanol for 1 h at 70°C. Samples were centrifuged and their supernatants were collected in separate vials. The sample extracts were dried using a vacuum concentrator (05-1-EV-02, Hanteul, Republic of Korea) and spray dryer (ODA-40, Seogang Eng., Republic of Korea), and kept in cold storage conditions (-80°C) till further use.

### Gut Microbiota Culture Conditions

Information about gut microbial strains used in the present study is listed in Table 1. We selected 37 different microbial strains across major phyla (Actinobacteria, Bacteroidetes, Firmicutes, Proteobacteria, and Verrucomicrobia) prevalent in human GIT toward gaining a comprehensive insight of their interaction and biotransformation of GT polyphenols. Further, we chose the strains based on their metabolic and genomic information, culturability, and availability at various culture collections for procurement. All bacterial strains were cultured anaerobically on brain–heart infusion (BHI) agar supplemented with 10% defibrinated horse blood (Kisan Biotech, Republic of Korea). Corresponding submerged cultures were grown in BHI medium supplemented with 0.005% cysteine. A flexible anaerobic chamber (Coy Laboratory Products) containing 10% CO_2_, 5% H2, and 85% N2 was used for culture maintenance and all other microbiological experiments under anaerobic conditions.

### In Vitro Screening for Gut Microbiota Mediated Biotransformation of GT Polyphenols

**Incubation of green tea polyphenols with gut bacteria.** Gut bacteria were precultured separately in BHI broth supplemented with 0.005% cysteine (5 ml) at 37°C for 48 h. Each preculture (70 μl) was then inoculated into 0.7 ml of fresh BHI broth containing catechin standards (0.2 mg/ml) and GT extracts (5 mg/ml) dissolved in dimethyl sulfoxide (DMSO) on a 96-well plate (Bioneer, Republic of Korea). We divided all gut bacteria into three groups with three different harvest times representing initial (lag phase), mid (exponential phase), and final (stationary phases) growth stages as the; (a) Fast group – 0 h, 6 h, 12 h; (b) Moderate group – 0 h, 12 h, 24 h; and (c) Slow group – 0 h, 24 h, 48 h. The aliquots (0.7 ml) of the incubation mixture were withdrawn in an anaerobic chamber after each of the three harvest periods, and immediately quenched in 0.7 ml of cold methanol. Subsequently, the harvested broth samples were placed on ice for 15 min and centrifuged (10 min,12,000 ×*g*, 4°C) to collect the supernatants. The culture supernatant broth extracts were stored at ultra-freeze conditions (-80°C) until analysis on UHPLC-LTQ-Orbitrap-MS/MS.

### UHPLC-LTQ-Orbitrap-MS/MS Analysis

Analytical samples for metabolite profiling were prepared by diluting culture supernatant broth extracts (0.2 ml) with HPLC-grade water (0.2 ml). The UHPLC-LTQ-Orbitrap-MS/MS system used was equipped with a Vanquish binary pump H system (Thermo Fisher Scientific, USA) coupled with an autosampler and a column compartment. Chromatographic separation was performed on a Phenomenex KINETEX C18 column (100 mm × 2.1 mm, 1.7μm; Torrance, USA) with an injection volume of 5 μl. The column temperature was set to 40°C, and the flow rate was 0.3 ml/min. The mobile phases consisted of 0.1% v/v formic acid in water (A) and 0.1% v/v formic acid in acetonitrile (B). The gradient parameters were set as described by Kwon *et al*. [19]. The MS data were collected in the range of 100–1500 m/z (under negative- and positive-ion mode) using an Orbitrap Velos ProTM system, which is combined with an Ion-Trap Mass Spectrometer (Thermo Fisher Scientific) coupled with a HESI-II probe. The probe heater and capillary temperatures were set to 300°C and 350°C, respectively. The capillary voltage was set to 2.5 kV in negative mode (positive mode, 3.7 kV).

### Bioactivity Assays

**Antioxidant assay.** The antioxidant activity was measured using the ABTS (2,2’-azino-bis (3-ethylbenzothiazoline-6-sulfonic acid) assay. Aliquots (200 μl) of the incubation mixture were evaporated using a speed-vacuum apparatus (Biotron, Korea) and dissolved in 100% methanol (0.5 mg/ml). ABTS (7 mmol) stock was dissolved in methanol and maintained at 60°C for 20 min until the absorbance of the solution reached 0.7 ± 0.02 at 750 nm as measured using a spectrophotometer (Spectronic Genesys 6, Thermo Electron, USA). The resulting solution was kept stable for the next 16 h and stored at 4°C. The assays were performed by adding 190 μl of ABTS solution to the sample extracts (10 μl, 0.5 mg/ml), and the resulting mixture was incubated for 7 min at 37°C in the dark. The absorbance was measured at a wavelength of 750 nm. The results were expressed as Trolox-equivalent activity concentrations (mM) and as the mean value of three analytical replicates.

### Total Phenol Content

The total phenolic content (TPC) assay was performed in two steps. First, the reaction mixture containing 20 μl of sample extract in 100% methanol (0.5 mg/ml) and 100 μl of 0.2N Folin-Ciocalteu’s phenol reagent was incubated for 5 min in the dark. Then 80 μl of 7.5% Na_2_CO_3_ was added, and the resulting reaction mixture was incubated for 60 min. Finally, the absorbance was measured at a wavelength of 750 nm. Assay results were expressed in terms of gallic acid equivalent for the activity (μg/ml) and as the mean value of three analytical replicates.

### Total Flavonoid Content

For the total flavonoid content (TFC) assay, the reaction mixtures contained 20 μl of the plant extract in 100%methanol (0.5 mg/ml), 20 μl of 0.1 N NaOH, and 160 μl of 90% diethylene glycol. The reaction mixture was incubated for 60 min and the absorbance was recorded at 405 nm. The results were expressed as naringin-equivalent activity concentrations (μg/ml). The data were expressed as the mean of three analytical replicates.

### Screening Growth Modulatory Effects of GT Polyphenols on Gut Microbiota

Gut bacteria were precultured separately in BHI broth supplemented with 0.005% cysteine (5 ml) at 37°C for 48 h. Each preculture (20 μl) was then inoculated into 0.2 ml of fresh BHI broth containing catechin standards and GT (0.5 mg/ml), dissolved in DMSO, in a 96-well plate in duplicate. Sample blanks containing 2% DMSO were used as controls. After inoculation, the microtiter plates were sealed with an adhesive sealing film to maintain an anaerobic atmosphere. The inoculation mixtures were incubated at 37°C for 48 h in a spectrophotometer, with optical density (O.D.) recorded automatically every 30 min at 600 nm, with a low-speed shaking for 5 s prior to each reading. This experiment was independently performed twice, with four replicates representing each strain. Growth curves were normalized with the background reads for media (0 h) subtracted from the reads of each incubated samples. Growth curves were constructed for each microbe separately by fitting data to the Baranyi model [20] using DMFit version 2.1 Excel add-in (Computational Microbiology Group of the Institute of Food Research, United Kingdom) to estimate the maximum specific growth rates (μ_max_) for gut microbes supplemented with GT and their metabolite extracts. Furthermore, the modulatory effects of flavonoid glycoside, flavonoid aglycone, and glucose were also measured. Their concentrations were also quantified in the GT extract using the UHPLC-LTQ-Orbitrap-MS/MS. Flavonoid glycosides were estimated in GT extracts using the authentic standards curves with kaempferol-3-O-galactoside (0.87-55.75 mM) and quercetin 3-glucoside (53.87-0.84 mM). Based on the flavonoids glycosides concentration in GT extracts, we treated each gut microbial culture with 0.003 mg/ml of GT extracts as described in previous section.

### Statistical Analysis

Peak area for the metabolites of interest were calculated based on the UHPLC-LTQ-Orbitrap-MS/MS datasets and their fold-change abundance were expressed using the heatmap. Significant differences in the antioxidant bioactivities of the sample extracts were tested using the analysis of variance (ANOVA) and Duncan’s multiple range test using PASW Statistics 18 (SPSS Inc., USA). Pearson’s correlation coefficient between the GT metabolites and antioxidant phenotypes were calculated using the PASW Statistics 18. The *p*-values for different metabolite-based clusters were determined using one-way ANOVA on STATISTICA software program (ver. 7.0; StatSoft, USA).

## Results

The current investigation explores two main biological questions. First, it examines the process by which gut bacteria transform GT polyphenols. Secondly, it investigates the impact of GT polyphenols and their derivative compounds on microbial growth in controlled in vitro settings. In the following sections, we elucidate each of these with experimental findings.

### Gut Bacteria Mediated Biotransformation of GT Polyphenols

**Fig. 2:**
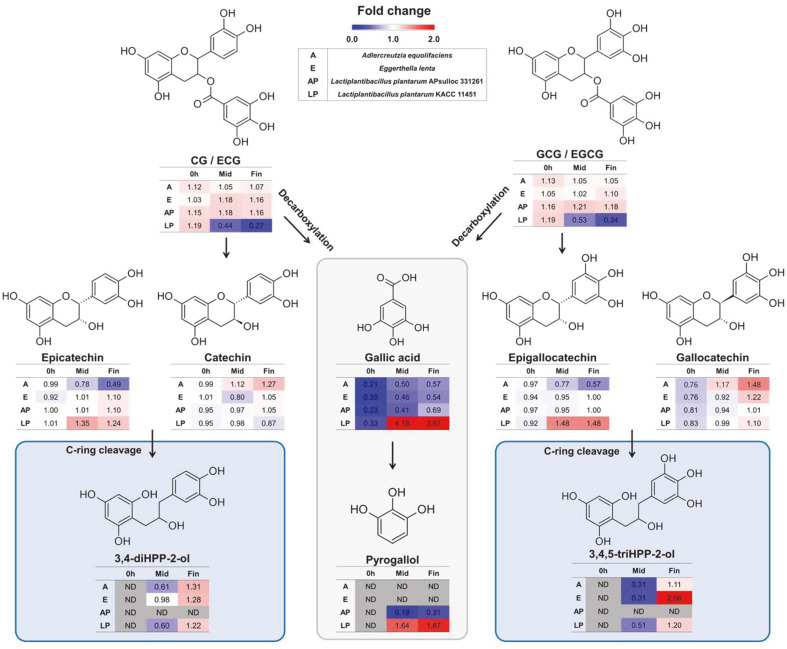
Proposed biotransformation mechanisms and heat-map showing the relative levels of major GT phenolics in spent media extracts from four gut bacteria. The colored squares indicate the fold changes (blue - to - red) normalized by the average abundance of the corresponding compounds in samples. Bacterial strain codes are the following: A: *Adlercreutzia equolifaciens*; E: *Eggerthella lenta*; AP: *Lactiplantibacillus plantarum* APsulloc 331261; LP: *Lactiplantibacillus plantarum* KACC11451.

**Fig. 3:**
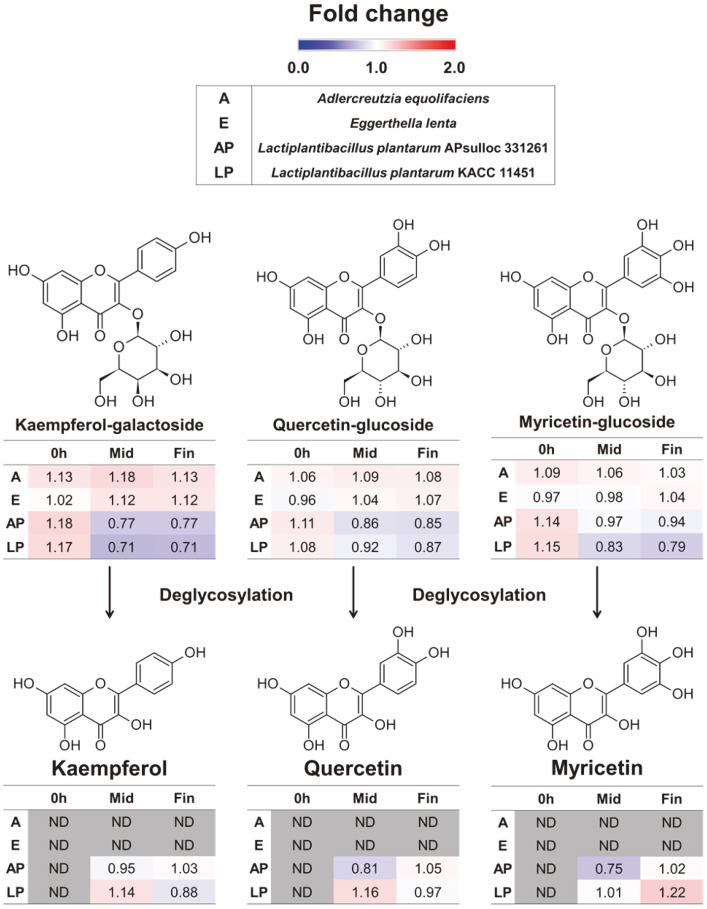
Proposed biotransformation pathways and heat-map showing the relative levels of major GT flavonoids in spent media extracts from four gut bacteria. The colored squares indicate the fold changes (blue - to - red) normalized by the average abundance of the corresponding compounds in samples. Bacterial strain codes are the following: A: *Adlercreutzia equolifaciens*; E: *Eggerthella lenta*; AP: *Lactiplantibacillus plantarum* APsulloc 331261; LP: *Lactiplantibacillus plantarum* KACC11451.

Under controlled in vitro conditions, we conducted a screening process involving 37 distinct human gut bacteria to assess their capacities for transforming GT polyphenols (Table 1). We incubated each bacterial species with GT extracts and selected standard compounds including catechin, epicatechin, and epigallocatechin gallate (EGCG). Based on the UHPLC-LTQ-Orbitrap-MS/MS analysis of the spent media extracts, we made observations regarding the catabolism of catechins in GT extracts. Notably, catechins were mainly degallolylated to diphenylpropanols, while its C-ring opening lead to the generation of phenylvalerolactones. In our investigation, we characterized 15 different catechin derivatives, of which we verified 10 compounds using authentic standards (Table 2). In our screening of 37 different gut bacteria to investigate the biotransformation of GT extracts and associated standard compounds (catechin, epicatechin, and EGCG), we observed that *Adlercreutzia equolifaciens* subsps. *Equolifaciens* KCTC 15235, *Eggerthella lenta* KCTC 3265, and *Lactiplantibacillus plantarum* KACC 11451 demonstrated the ability to perform C-ring cleavage. However, another strain *L. plantarum* APsulloc 331261 used in this study was not able to perform C-ring cleavage of GT catechins. However, both *L. plantarum* strains were noteworthy for their abilities to hydrolyze catechin galloyl esters, resulting in the production of gallic acid and its pyrogallol derivatives (Fig. 2). Additionally, these strains also carried out the production of deglycosylated flavonoid aglycones (Fig. 3).

### Biotransformation of GT Polyphenols in Different Growth Stages of Gut Microbiota

We observed that four gut microbial species & strains including *A. equolifaciens* (abbrev. A), *E. lenta* (abbrev. E), *L. plantarum* APsulloc 331261 (abbrev. AP), and *L. plantarum* KACC 11451 (abbrev. LP) were primarily involved in the biotransformation of the GT polyphenols (Fig. 2). For LP, we observed multifold levels of gallic acid (4.19X & 3.67X) and pyrogallol (1.64X & 1.87X) during the mid (exponential phase) and final (stationary phase) growth stages, respectively, compared to others species/ strains used in the study. Among the C-ring cleavage reaction product of GT polyphenols, the relative abundance of 3,4-diHPP-2-ol (1-(3,4-dihydroxyphenyl)-3-(2,4,6-trihydroxyphenyl) propan-2-ol) was significantly higher during the final growth stages for gut bacteria A, E, and LP. However, this particular C-ring cleavage derivative of catechin was not detected in AP. Another C-ring cleaved derivative from Epigallocatechin/ Gallocatechin precursors was 3,4,5-triHPP-2-ol also displayed similar trends among the four species with significantly higher titers toward the stationary phase. Notably, pyrogallol and both the C-ring cleaved derivatives (3,4-diHPP-2-ol and 3,4,5-triHPP-2-ol) were not detected from the time control samples harvested at 0 h.

Flavonoid glycosides including kaempferol galactoside, quercetin glucoside, and myricetin glucoside were primarily biotransformed to their deglycosylated derivatives (Fig. 3). Notably, flavonoid glycosides including kaempferol-galactoside, quercetin-glucoside, and myricetin-glucoside significantly decreased during the mid-and final growth stages for both the firmicutes including *Lactobacillus* strains used in the study. However, the relative levels of flavonoid glycosides remain roughly unchanged for Actinobacteria (*A. equolifaciens* and *E. lenta*) throughout the course of incubation. On the other hand, their flavonoid aglycone derivatives (kaempferol, quercetin, and myricetin) were only detected during the mid- and final growth stages selectively for the two *Lactiplantibacillus* strains (AP and LP) representing the phylum Firmicute (Fig. 3).

### Antioxidant Bioactivities of GT Extracts Following Gut Microbiota Mediated Biotransformation

**Fig. 4:**
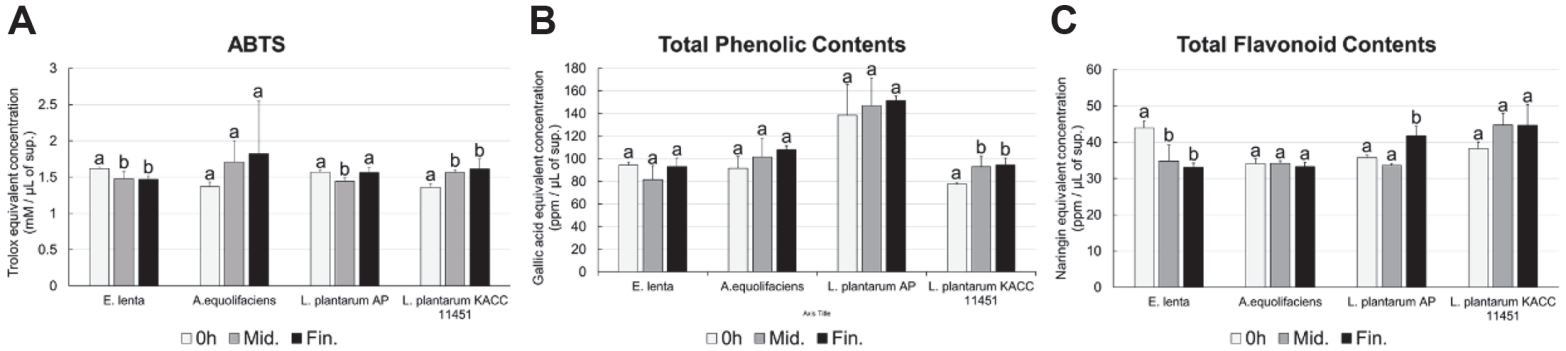
Bioactivity phenotypes for gut microbial spent media extracts following GT treatment. The bar graphs represent, (**A**) antioxidant activity, (**B**) total phenolic contents, and (**C**) total flavonoid content, for gut microbial cultures. The bar colors represent different time points - white color: 0 h; gray color: mid harvest point; black color: final harvest point. All values are expressed as the average of three biological replicates with standard deviation. The bar graph denoted by the same letter indicates absence of statistical differences, according to Duncan’s multiple range test (*p* < 0.05).

ABTS radical scavenging assay showed that the antioxidant bioactivity for *L. plantarum* KACC 11451 (LP) culture mediate extracts with GT polyphenols increased significantly during the later stages of incubation compared to control. For rest of the three cultures (A, E, and AP), we did not observe any significant changes in ABTS bioactivity (Fig. 4A). Considering the total phenolic contents, we observed similar trends for LP with significantly higher levels of phenolic compounds at mid and final growth phases compared to 0 h (Fig. 4B). Cultures A and AP also displayed marginally increased levels of total phenolics except E. Intriguingly, higher total flavonoid levels were recorded for both the *Lactiplantibacillus* strain (AP and LP) compared to control cultures harvested at 0 h (Fig. 4C). In contrast, total flavonoids were marginally decreased in *E. lenta* (E) and remain unchanged for *A. equolifaciens* (A).

### Pearson’s Correlation between the Relative Levels of Biotransformed GT Polyphenols and the Antioxidant Bioactivities for Gut Microbiota Culture Extracts

**Fig. 5:**
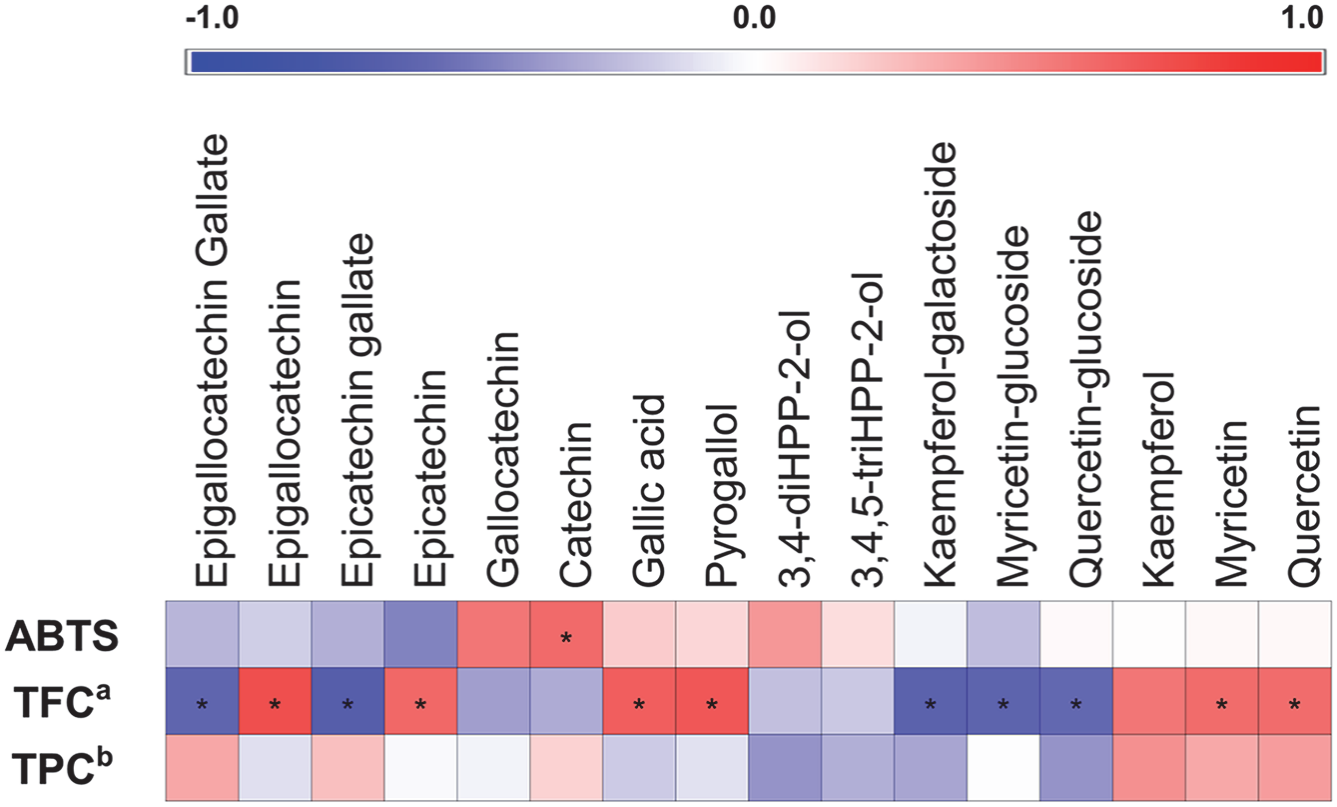
Pearson’s correlation analysis between bioactivity phenotypes and GT metabolites biotransformed in gut bacteria cultures. Herein, the bioactivity phenotypes including antioxidant activity (ABTS), total flavonoid contents (TFC), and total phenolic contents (TPC) were correlated with GT polyphenols and their derivatives. Strength of Pearson’s correlation coefficient values (*r*) between GT metabolites and bioactivity phenotypes is represented with heat-map. Red and blue a indicate positive (0 < *r* < 1) and negative (-1 < *r* < 0) correlation, respectively. (*p < 0.05, ^a^TFC represents the Total Flavonoid Contents; ^b^TPC represents the Total Phenolic Contents).

Pearson’s coefficients (r) were estimated for both the positive (0 < r < 1) and negative (-1 < r < 0) statistical correlations between the GT polyphenols & its derivative compounds and bioactivity phenotypes of gut microbiota cultures (Fig. 5). Among the GT polyphenols, only catechin displayed strong positive correlations with antioxidant ABTS bioactivity while the remaining derivative compounds including gallocatechin, gallic acid, 3,4-diHPP-2-ol, and 3,4,5-triHPP-2-ol showed positive but non-significant correlations. GT flavonoids including EGC, EC, and flavonols like kaempferol, myricetin, and quercetin were strongly correlated with TFC bioactivity. In addition, phenolic compounds like gallic acid and pyrogallol were also correlated strongly positive with TFC. Similar observations were valid between TPC and the relative levels of most GT phenolic compounds including kaempferol, myricetin, quercetin and flavonoids like EGCG and ECG (Fig. 5).

### Growth Modulatory Effects of GT Polyphenols on Gut Microbiota

**Fig. 6:**
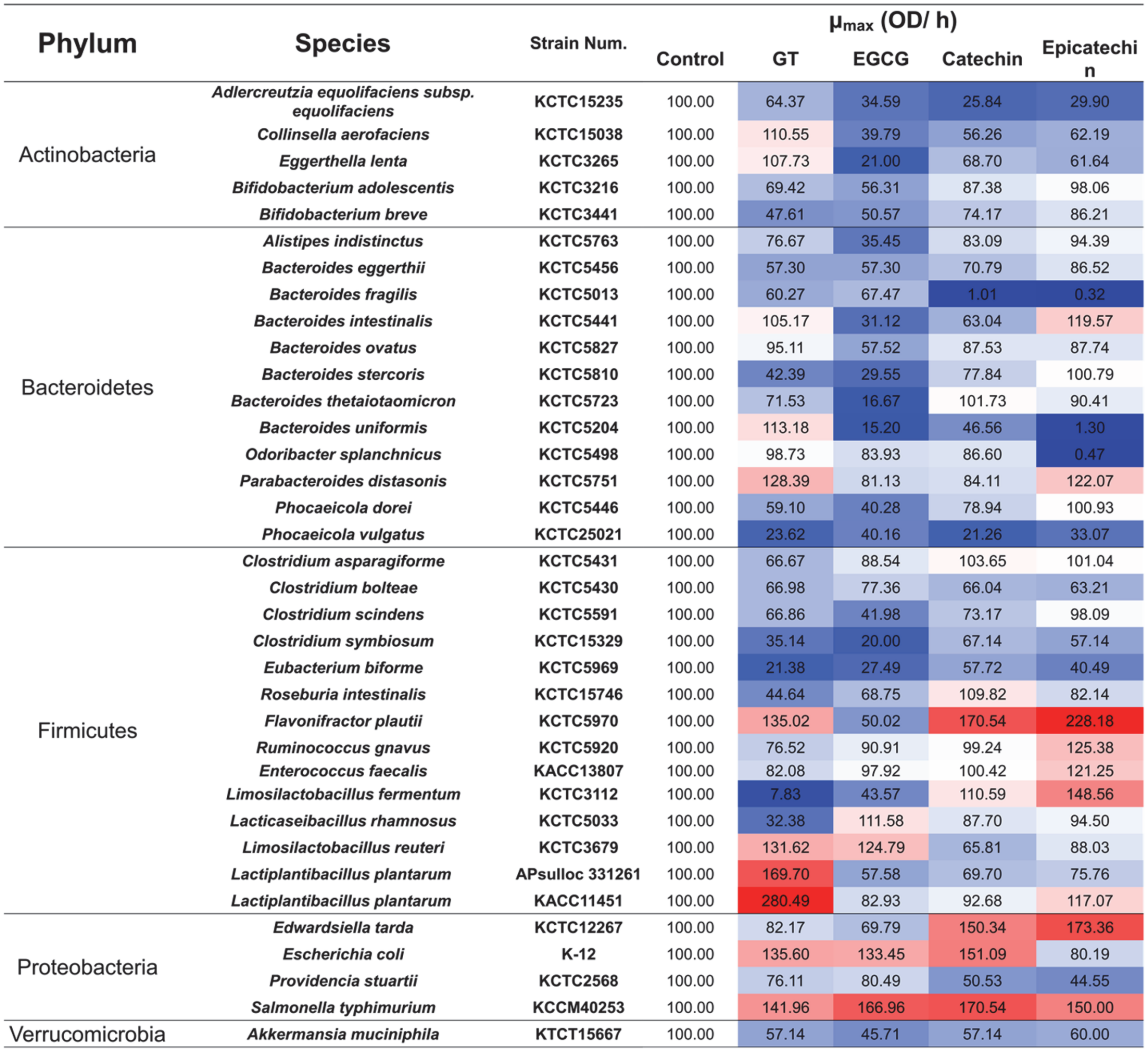
The growth modulatory effects of green tea (GT) polyphenols and derivatives on growth rates (μ_max_) of gut microbes used in this study. The variation in growth rates are indicated with heap-map and the values in colored squares represent the fold changes (blue - to - red) normalized with growth rate for corresponding control cultures.

GT extracts and related standard compounds (catechin and epicatechin) were screened for their growth modulatory effects on 37 different gut bacteria. Most notably, growth of firmicutes and proteobacteria were stimulated by GT treatments compared to those observed in the respective control groups (Fig. 6). Among the firmicutes, GT polyphenols significantly enhanced the growth of *Limosilactobacillus* and *Lactiplantibacillus* species, but suppressed the growth of *Clostridium* species compared to control groups. Growth of selected Proteobacteria including *Escherichia coli* and *Salmonella typhimurium* was also enhanced by GT polyphenol treatment. Further, gut microbes belonging to Bacteroidetes and Actinobacteria phyla were significantly inhibited by GT extracts and associated standard compounds. At the same time, growth of some Bacteroidetes including *B. uniformis* and *P. distasonis* were significantly enhanced by GT polyphenols.

**Fig. 7:**
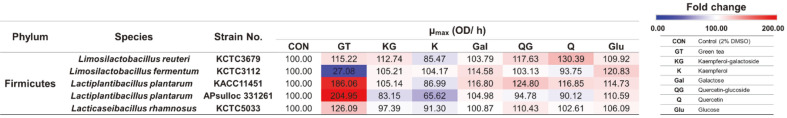
The growth modulatory effect of green tea (GT) extracts and their major flavonoids on selected gut microbe growth rates (μ_max_). The values in heat-map represent the fold changes (blue - to - red) values for growth rates normalized with corresponding control cultures.

GT flavonoid EGCG inhibited most gut bacteria except Lacticaseibacillus rhamnosus, *Limosilactobacillus reuteri*, and selected Proteobacteria (*E. coli* and *S. typhimurium*). Other major GT flavonoids including catechin and epicatechin showed different and contrasting effects on gut bacteria growth. Higher growth of firmicutes including *Flavonifractor plautii*, *Rumonococcus gnavus*, *Enterococcus faecalis*, and *Limosilactobacillus fermentum* was recorded when treated with epicatechin compared to catechin treated cultures. However, both catechin and epicatechin promoted significantly higher growth of Proteobacteria including *Edwardsiella tarda* and *S. typhimurium* (Fig. 6). Further, we observed a considerably higher growth rates for *Lactiplantibacillus* species treated with GT flavonoid glycosides however their aglycones derivatives inhibited the cultures (Fig. 7). We recorded higher growth rates for *L. rhamnosus*, *L. reuteri*, the two *L. plantarum* strains media-fed with GT extracts, compared to control. In contrast, cultures with deglycosylated forms of GT flavonoids, especially kaempferol, displayed a significantly lower growth compared to control. Intriguingly, we recorded markedly higher growths for all five *Lactobacillus* species fed with monosaccharide sugars (galactose and glucose) in the growth medium.

## Discussion

Polyphenols rich in GT are recognized as xenobiotics by human body and they reach colon almost unmetabolized. However, polyphenol compounds are chemically transformed in the gut microenvironment to various easily assimilable bioactive derivatives [18]. Human gut microbes have an extensive capacity to metabolize and biotransform phytochemicals through employing diverse metabolic pathways [21, 22]. However, different gut bacteria display varied abilities and preferences to metabolize plant polyphenols and this determines their different bioavailability and bio-efficacy in humans. Hence, we argue that characterizing different species of human gut microbiota which metabolize nutritional phytochemicals, including GT polyphenols, is crucial for designing effective nutritional interventions in humans.

Present study explores the metabolic fate of GT polyphenols using indigenously developed in vitro screening method which involve media-feeding the GT extracts to 37 gut microbiota cultures. Biotransformation of GT compounds was monitored using the time-correlated analysis of the spent media extracts corresponding to different growth stages for different gut microbiota using UHPLC-LTQ-Orbitrap-MS/MS analysis. We primarily focused and validated the biotransformation of catechins, one of the most abundant & characterized GT polyphenols, using standard compounds. Catechins are primarily catabolized by gut microbiota using degalloylation, C-ring opening, and A-ring fission mechanisms which results in the production of diphenylpropanols, phenylvalerolactones, and phenylvaleric acids, respectively [12]. In congruence with the previous studies, we observed that Actinobacteria (*A. equolifaciens* and *E. lenta*) were able to catabolize GT catechins through C-ring opening reaction [23]. Among the firmicutes, we observed a selective metabolic preference for C-ring opening reaction by different *Lactiplantibacillus* strains. Very few and selected strains of *L. plantarum* are reported to catabolize GT compounds using C-ring opening mechanisms which suggest an inherent metabolic diversity among the firmicutes [24]. Further, we also noted subsequent biotransformation mechanisms for GT metabolites by *L. plantarum* which include hydrolysis of galloyl esters to gallic acid and flavonoid glycosides to aglycone derivatives.

Mechanistically, galloylated flavan-3-ols esters are readily hydrolyzed by gut bacterial esterases to gallic acid, which is further decarboxylated to its pyrogallol derivatives [25]. In accordance with this established pathway, we recorded a linear rise in the relative abundance of gallic acid and its pyrogallol derivatives for most Actinobacteria species during the mid and late growth stages. As observed in this study, *L. plantarum* is reported to carry out both the galloyl-esterase and decarboxylase bioconversion of polyphenol substrates in tandem [26]. Since, these polyphenol derivatives were only detected during the later stages of gut microbiota incubation, we argue that their bioavailability is essentially facilitated by gut microbiome following GT consumption. Most plant flavonoids exist as C- and/or O-glycosides which necessarily need to be deglycosylated for effective assimilation by human digestive system [17]. This deglycosylation of flavonoid glycosides is primarily carried out by gut microbiota phyla Bacteroides and Firmicutes [27]. For GT polyphenols in this study, we observed a linear decrease in the relative levels of flavonoid glycosides and corresponding rise in their aglycone derivatives, compared to control (0 h culture extracts). These observation supports the β-glucosidase mediated deglycosylation mechanisms for flavonoid glycosides in GT extracts [17].

Antioxidant and health functions of GT are mainly attributed to its polyphenol contents and their derivative compounds released following gut microbiota mediated biotransformation [28]. We recorded a linear increase in the antioxidant bioactivities and total phenolics for the spent media extracts from cultures added with GT extract and standard catechins. Though, total flavonoids were decreased which indicate their biotransformation to aglycone derivatives (Fig. 4). We further substantiated this using Pearson’s correlation coefficients for the GT polyphenols and associated biotransformed derivatives with bioactivity phenotypes for spent media extracts. In congruence with previous study, we observed strong positive and linear correlation between C-ring cleaved and degallolylated polyphenol derivatives [11, 29]. Enhanced antioxidant activities are reported in tea leaves following tannase mediated hydrolysis of tannin polyphenols and bioconversion to gallic acid derivatives [30]. Hence, we argue that GT polyphenol biotransformation by gut microbiota, most notably by *L. plantarum* species, might significantly contribute to GT polyphenol bioavailability and health effects in host. A number of studies have reported the gut microbiota mediated biotransformation of dietary polyphenols and how this significantly enhances their antioxidant functions which helps mitigating the inflammatory stress and ROS (Reactive Oxygen Species) generation in host [31-35].

Another important aspect of this study deals with growth modulatory effects of GT polyphenols and their biotransformed derivatives on gut microbiota. Most interestingly, GT polyphenols and their derivative compounds supported the higher growth rates for genus *Lactobacillus* (Phylum: Firmicutes; Class: Bacilli). Considering its health implications, *Lactobacillus* is a well-known probiotic and a most promising candidate for boosting growth of endogenous beneficial microbes, gut health, and immune defense mechanisms against pathogenic bacteria in humans [36-38]. Reportedly, fecal *L. reuteri* colonizes numerous mammals and produces antimicrobial molecules, inhibits the colonization of pathogenic microbes, strengthens the intestinal barrier, and ameliorates inflammatory diseases [39]. Hence, any stimulation of beneficial *Lactobacillus* species with GT polyphenols can be positively correlated with functional aspects of GT consumption. GT polyphenols and their derivatives did not influence the growth of most Clostridium species used in this study except *C. asparagiforme* whose growth was marginally enhanced by GT catechin and epicatechin (Fig. 6). Most bacteria in *Clostridium* genus are considered pathogenic which acts by biotransformation of some ingested compounds to harmful products including N-nitroso compounds or aromatic steroids in gastrointestinal tract (GIT) [40]. In contrast, some *Clostridium* spp. are also reported to function as probiotic and maintain intestinal homeostasis [41]. Among the phylum Proteobacteria, GT polyphenols and their derivatives supported the higher growth of opportunistic pathogens (*Escherichia coli* and *Salmonella typhimurium*). Though we observed a significantly higher growth of these two Proteobacteria, it was largely independent of the GT biotransformation as both the GT extracts as well as catechins (EGCG, catechin, and Epicatechin) effected comparable growth. Two major phyla representing human gut microbiome are Bacteroidetes and Actinobacteria, and we noted that growth of most species among them were either not influenced or inhibited by GT metabolites (Fig. 6). Bacteroidetes represent a considerably large (~20-40%) population of human colonic bacteria and confers important metabolic, immunologic, and defensive functions in GIT [42]. Most notably, the growth of selected Bacteroidetes including *B. uniformis* and *P. distasonis* were stimulated by GT polyphenols. This is important as infant colonic *B. uniformis* exhibits the ability to boost anti-inflammatory cytokine production and ameliorates metabolic and immune dysfunction [43]. Another species, *P. distasonis* has an ambivalent effect, with several studies describing it’s pro-inflammatory as well as anti-inflammatory effects depending on certain conditions [44, 45]. Altogether, we observed mixed effects for GT polyphenols in promoting or inhibiting human gut microbiota which can largely be ascribed to their selective antimicrobial functions described previously [46]. Notably, the gut bacterial species which perform C-ring cleavage were better survived with higher growth rates compared to those which couldn’t perform this bioconversion (Fig. S1). Considering the effects of deglycosylation of GT flavonoids on gut bacteria, we observed selectively higher growth for most *Lactobacillus* species supplied with flavonoid glycosides (Kaempferol-galactoside and Quercetin-glucoside) compared to their aglycone derivatives. This can be ascribed to the β-glucosidase activity of *Lactobacillus* species which readily hydrolyzed flavonoid glycosides to their aglycone forms, and release monosaccharide sugars which serve as additional C-source for growing bacteria [47]. This was substantiated by the comparable rise in growth rates of all *Lactobacillus* species supplied with monosaccharide sugars, galactose and glucose (Fig. 7). In addition, relatively higher antimicrobial effects of flavonoid aglycones can also be attributed to the reduced growth for *Lactobacillus* species. Reportedly, flavonoid aglycones are more potent antioxidant and antibacterial compounds compared to their glycosylated precursors [13]. Therefore, we suggested that many synergistic factors work together and contribute to the growth-promoting effects of GT polyphenol metabolites.

In conclusion, the study highlights the likely nature of ‘metabolite–microbe’ interaction, specifically the biotransformation of GT metabolites by gut microbiota and the impact of GT metabolite derivatives on microbial growth. *Lactobacillus* mediated C-ring opening for GT polyphenols was the most prominent biotransformation mechanism generating antioxidant derivatives including 3,4-diHPP-2-ol and 3,4,5-triHPP-2-ol. Two other biotransformation mechanisms prevalent among *Lactiplantibacillus* cultures were degalloylation of GT catechins and deglycosylation of flavonoid glycosides. Reciprocally, we substantiated the growth modulatory and probiotic effects of GT polyphenols and derivatives on gut microbiota. Though this study offers insights into the gut microbiota mediated biotransformation of GT metabolites and their interaction with gut bacteria themselves, it will be noteworthy to test and validate these results using in vivo experimental models. However, we strongly believe that the in vitro screening methodology used in this study can still be leveraged to examine ‘microbe-metabolite’ interactions for a large number of unexplored dietary phytochemicals and drugs metabolized by gut microbiome.

## Supplemental Materials

Supplementary data for this paper are available on-line only at http://jmb.or.kr.

## Figures and Tables

**Table 1:** Information about 37 Gut microbes used in this study.

Phylum	Class	Genus	Species	Strain No.	Source
Actinobacteria	Coriobacteriia	Adlercreutzia	*Adlercreutzia equolifaciens* subsp. *equolifaciens*	KCTC15235	Human faeces
		Collinsella	*Collinsella aerofaciens*	KCTC15038	Human faeces
		Eggerthella	*Eggerthella lenta*	KCTC3265	Rectal tumor
		Bifidobacterium	*Bifidobacterium adolescentis*	KCTC3216	Human intestine
			*Bifidobacterium breve*	KCTC3441	Human faeces
Bacteroidetes	Bacteroidia	Alistipes	*Alistipes indistinctus*	KCTC5763	Human faeces
		Bacteroides	*Bacteroides eggerthii*	KCTC5456	Human faeces
			*Bacteroides fragilis*	KCTC5013	Appendix abscess
			*Bacteroides intestinalis*	KCTC5441	Human faeces
			*Bacteroides ovatus*	KCTC5827	Unknown
			*Bacteroides stercoris*	KCTC5810	Human faeces
			*Bacteroides thetaiotaomicron*	KCTC5723	Human faeces
			*Bacteroides uniformis*	KCTC5204	Human faeces
		Odoribacter	*Odoribacter splanchnicus*	KCTC5498	Abdominal abscess
		Parabacteroides	*Parabacteroides distasonis*	KCTC5751	Unknown
		Phocaeicola	*Phocaeicola dorei*	KCTC5446	Human faeces
			*Phocaeicola vulgatus*	KCTC25021	Human faeces
Firmicutes	Clostridia	Clostridium	*Clostridium asparagiforme*	KCTC5431	Human faeces
			*Clostridium bolteae*	KCTC5430	Human faeces
			*Clostridium scindens*	KCTC5591	Human faeces
			*Clostridium symbiosum*	KCTC15329	Unknown
		Eubacterium	*Eubacterium biforme*	KCTC5969	Human faeces
			*Eubacterium rectale*	KCTC5835	Human faeces
		Flavonifractor	*Flavonifractor plautii*	KCTC5970	Unknown
		Roseburia	*Roseburia intestinalis*	KCTC15746	Infant faeces
		Ruminococcus	*Ruminococcus gnavus*	KCTC5920	Human faeces
	Bacilli	Enterococcus	*Enterococcus faecalis*	KACC13807	Unknown
		Lactobacillus	*Limosilactobacillus fermentum*	KCTC3112	Fermented beets
			*Lacticaseibacillus rhamnosus*	KCTC5033	Clinical source
			*Limosilactobacillus reuteri*	KCTC3679	Unknown
			*Lactiplantibacillus plantarum*	APsulloc 331261	Green tea
			*Lactiplantibacillus plantarum*	KACC11451	Pickled cabbage
Proteobacteria	Gammaproteobacteria	Edwardsiella	*Edwardsiella tarda*	KCTC12267	Human faeces
		Escherichia	*Escherichia coli*	K-12	Human faeces
		Providencia	*Providencia stuartii*	KCTC2568	Unknown
		Salmonella	*Salmonella typhimurium*	KCCM40253	Clinical source
Verrucomicrobia	Verrucomicrobiae	Akkermansia	*Akkermansia muciniphila*	KTCT15667	Human faeces

**Table 2:** Mass spectral characteristics of green tea (GT) polyphenols and their derivative compounds annotated using UHPLC-LTQ-Orbitrap-MS/MS analysis.

RT^a^ (min)	Tentative metabolites	*m/z* ^b^		M.W.^c^	Mol. Formula	Delta ppm	Fragment patterns	Ref.^d^
		[M-H]^-^	[M+H]^+^					
*Catechin derivatives*								
4.43	Epigallocatechin Gallate	457.0756	-	458	C_22_H_18_O_11_	-4.4	-	STD^e^
4.85	Epicatechin gallate	441.0817	-	442	C_22_H_17_O_10_	-2.2	441>289>245>203	STD
1.67	Gallocatechin	305.0659	307.0797	306	C_15_H_14_O_7_	-5.2	305>179>164>120	STD
3.36	Epigallocatechin	305.0648	307.0794	306	C_15_H_14_O_7_	-5.1	305>261, 179>163>120	STD
3.68	Catechin	289.0702	291.0867	290	C_15_H_14_O_6_	1.4	289>245>203>175	STD
4.22	Epicatechin	289.0702	291.0867	290	C_15_H_14_O_6_	1.4	289>245>203>175	STD
3.45	1-(3′,4′,5'-trihydroxyphenyl)-3-(2′′,4′′,6′′-trihydroxyphenyl) propan-2-ol(3,4,5-triHPP-2-ol)	307.0822	-	308	C_15_H_16_O_7_	-0.3	-	[49]
4.26	1-(3′,4′-dihydroxyphenyl)-3-(2′′,4′′,6′′-trihydroxyphenyl) propan-2-ol(3,4-diHPP-2-ol)	291.0875	293.1011	292	C_15_H_15_O_6_	0.3	291>247>205>177, 135	[49]
0.86	Gallic acid	169.0134	171.0283	170	C_7_H_6_O_5_	-5.0	169>125>97, 81	STD
1.22	Pyrogallol	125.0253	127.0387	126	C_6_H_6_O_3_	-4.2	125>107	STD
*Flavonoid derivatives*								
5.46	Myricetin	317.0288	319.0429	318	C_15_H_10_O_8_	-4.6	317>179>151>107	STD
5.98	Quercetin	301.0338	303.048	302	C_15_H_10_O_7_	-5.4	301>179>151>107	STD
6.52	Kaempferol	285.0388	287.0534	286	C_15_H_10_O_6_	-5.8	285>151>106>65	STD
4.98	Quercetin-glucoside	463.0868	465.1033	464	C_21_H_20_O_12_	-3.0	463>301>179>151	STD
5.12	Kaempferol-galactoside	447.0917	449.1088	448	C_21_H_20_O_11_	-3.6	447>284>255>227,221	STD

^a^Retention times

^b^Mass detected in the experiment

^c^Olecular weight

^d^Reference

^e^STD, mass spectrum was consistent with that of standard compound
