# Supplementary material for: Bidirectional Interactions between Green Tea (GT) Polyphenols and Human Gut Bacteria
Source: J Microbiol Biotechnol. 2023 Jul 12;33(10):1317–28. doi: 10.4014/jmb.2306.06014 (PMC10619559; doi:10.4014/jmb.2306.06014)
Supplement: Supplementary file 1 [file jmb-33-10-1317-supple.pdf]

J. Microbiol. Biotechnol.
https://doi.org/10.4014/jmb.2306.06014

Supplementary Figure

Bidirectional Interactions between Green Tea (GT) Polyphenols and Human Gut Bacteria

Se Rin Choi [1¶], Hyunji Lee [1¶], Digar Singh[1], Donghyun Cho [2], Jin-Oh Chung [2], Jong-Hwa Roh [2], Wan-Gi Kim [2], Choong Hwan Lee[1, 3*]

[¶] These authors contributed equally to this work.

[1] Department of Bioscience and Biotechnology, Konkuk University, Seoul, Republic of Korea

[2] Amorepacific R&I Center, Yonggu-daero, Yongin, Republic of Korea

[3] Research Institute for Bioactive-Metabolome Network, Konkuk University, Seoul, Republic of Korea

*Corresponding author:

Email: chlee123@konkuk.ac.kr

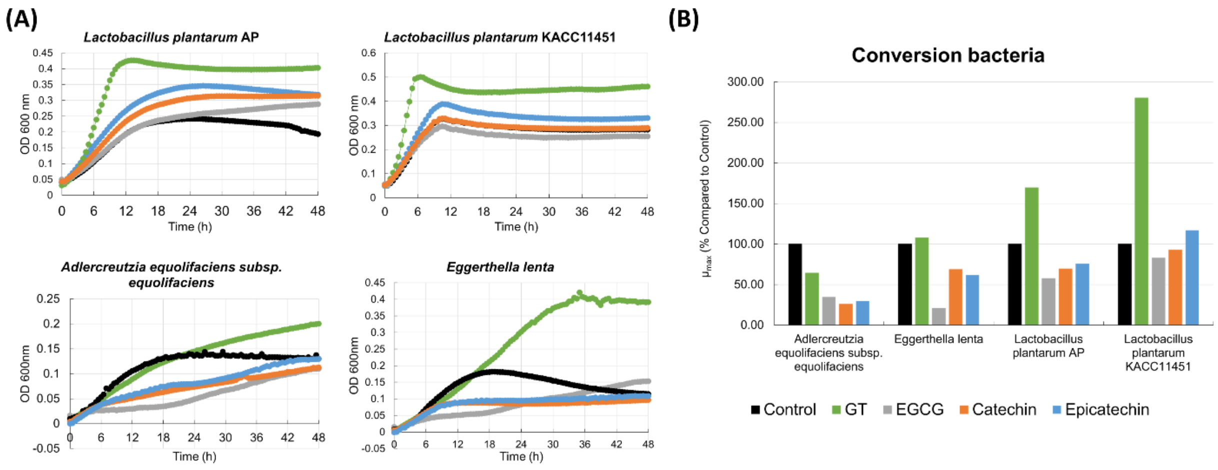

Figure S1. Effect of major green tea (GT) phenolics on selected gut bacteria. (A) line graph representing the growth curves, and **(B)** bar graph showing the growth rates (μ_{max}) for four major gut bacteria performing biotransformation of GT compounds compared to control. The color for line and bar graphs represents the following: **black color** – control group; **green color** – bacteria media-fed with GT; **orange color** – bacteria media-fed with GT catechin; **blue color** – bacteria media-fed with GT epicatechin; **gray color -** bacteria media-fed with GT EGCG).
